# Evaluation of *Brachypodium distachyon* L-Tyrosine Decarboxylase Using L-Tyrosine Over-Producing *Saccharomyces cerevisiae*

**DOI:** 10.1371/journal.pone.0125488

**Published:** 2015-05-21

**Authors:** Shuhei Noda, Tomokazu Shirai, Keiichi Mochida, Fumio Matsuda, Sachiko Oyama, Mami Okamoto, Akihiko Kondo

**Affiliations:** 1 Biomass Engineering Program, RIKEN, Yokohama, Kanagawa, Japan; 2 Department of Bioinformatic Engineering, Graduate School of Information Science and Technology, Osaka University, Suita, Osaka, Japan; 3 Department of Chemical Science and Engineering, Graduate School of Engineering, Kobe University, Kobe, Japan; CNR, ITALY

## Abstract

To demonstrate that herbaceous biomass is a versatile gene resource, we focused on the model plant *Brachypodium distachyon*, and screened the *B*. *distachyon* for homologs of tyrosine decarboxylase (TDC), which is involved in the modification of aromatic compounds. A total of 5 candidate genes were identified in cDNA libraries of *B*. *distachyon* and were introduced into *Saccharomyces cerevisiae* to evaluate TDC expression and tyramine production. It is suggested that two TDCs encoded in the transcripts Bradi2g51120.1 and Bradi2g51170.1 have L-tyrosine decarboxylation activity. Bradi2g51170.1 was introduced into the L-tyrosine over-producing strain of *S*. *cerevisiae* that was constructed by the introduction of mutant genes that promote deregulated feedback inhibition. The amount of tyramine produced by the resulting transformant was 6.6-fold higher (approximately 200 mg/L) than the control strain, indicating that *B*. *distachyon* TDC effectively converts L-tyrosine to tyramine. Our results suggest that *B*. *distachyon* possesses enzymes that are capable of modifying aromatic residues, and that *S*. *cerevisiae* is a suitable host for the production of L-tyrosine derivatives.

## Introduction

Plants produce various kinds of compounds containing aromatic residues via secondary metabolite pathways, such as the phenylpropanoid biosynthesis pathway [1–7]. Although a number of plant genes involved in the modification of aromatic residues have been identified, the majority of plant genomes have not been sequenced due to their large sizes compared to those of microbes and are expected to contain numerous novel and unidentified genes.

*Brachypodium distachyon* is a model plant for cereal crops, such as barley and wheat, and is often used for biological characterization of grass biomass due to its short life cycle, small size, simple transformation procedure and small genome size [8]. Recently, full-length cDNA libraries of *B*. *distachyon* were constructed and have been made publically available [9]. However, there are few reports concerning the characterization and application of genes and proteins derived from *B*. *distachyon*.

The yeast *S*. *cerevisiae* has been widely studied and is commonly used as a model eukaryote. Various heterogeneous genes have been functionally characterized using *S*. *cerevisiae* as a host strain [10–13]. Genetically modified *S*. *cerevisiae* strains have also been used in the fermentation industry to produce various compounds, including fuels and organic acids [13, 14]. *S*. *cerevisiae* has also been used as host for the biosynthesis of aromatic compounds. For example, Kim et al. [15] reported 2-phenylethanol production via the Ehrlich pathway, and Vannelli et al. [16] demonstrated *p*-hydroxycinnamic acid production using a cytochrome P-450-expressing strain of *S*. *cerevisiae*. Koopman et al. successfully produced flavonoid naringenin using genetically engineered *S*. *cerevisiae* [17].

**Fig 1:**
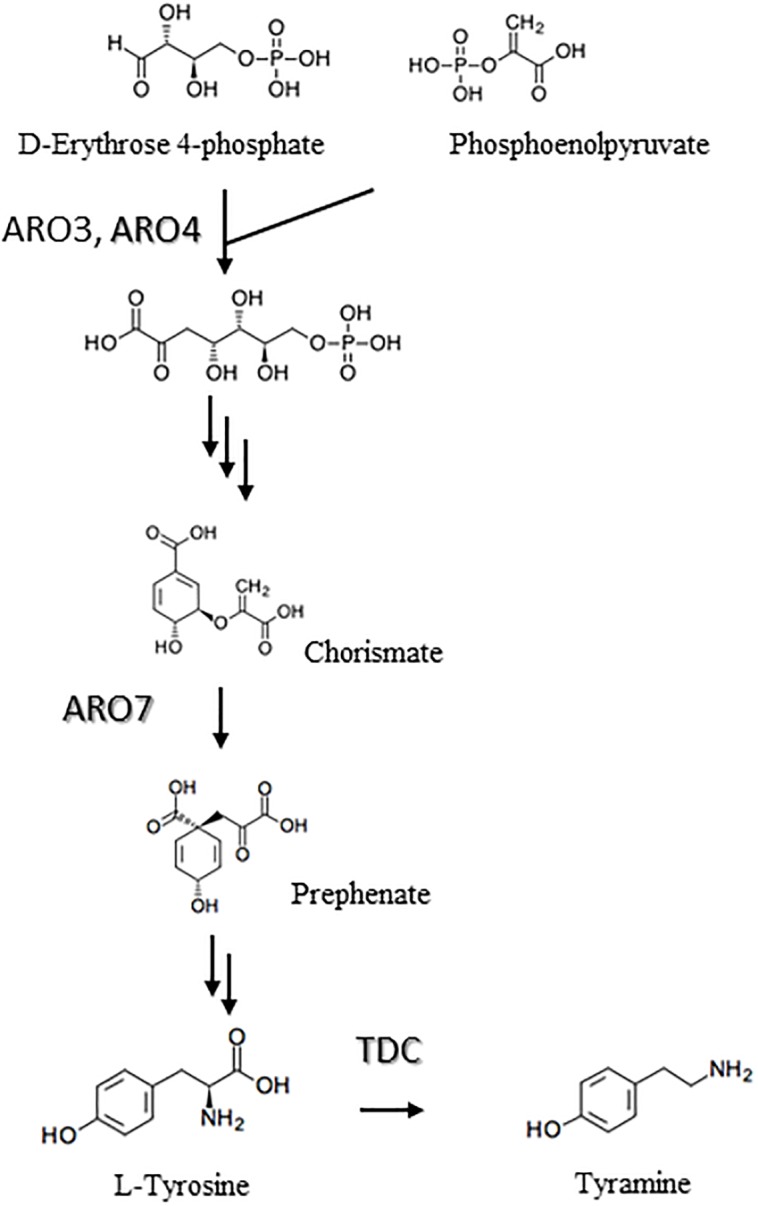
Proposed biosynthesis pathway for tyramine (ARO3, ARO4; 3-deoxy-D-heptulosonate-7-phosphate synthase: ARO7; chorismate mutase: TDC; L-tyrosine decarboxylase). ARO3, ARO4 and ARO7 are derived from *S*. *cerevisiae*, whereas TDC is originated from *B*. *distachyon*.

The shikimate pathway is a metabolic route for the biosynthesis of aromatic amino acid in microorganisms. The first reaction in this pathway involves the stereo-specific condensation of erythrose-4-phosphate (E4P) and phosphoenolpyruvate (PEP) to 3-deoxy-D-heptulosonate-7-phosphate (DAHP) in a reaction catalyzed by DAHP synthase (Fig 1) [18]. In *S*. *cerevisiae*, DAHP synthase is encoded by the *ARO3* and *ARO4* genes, and the corresponding proteins, ARO3 and ARO4, are strongly regulated by L-phenylalanine and L-tyrosine, respectively, which are produced in this pathway [19]. According to a report by Helmstaedt et al. [20], a single serine-to-alanine substitution in ARO4 at position 195 impairs L-tyrosine sensitivity, leading to deregulation of ARO4. The conversion of chorismate to phenylpyruvate (PPA) by chorismate mutase is another step regulating aromatic amino acid productivity in this pathway [18]. *S*. *cerevisiae* chorismate mutase is encoded by *ARO7* and its activity is inhibited by L-tyrosine and L-tryptophan; however, the substitution of glycine with serine at position 141 generates L-tyrosine-insensitive ARO7 [19]. Although these findings indicate that enzymes involve in amino acid biosynthesis in *S*. *cerevisiae* can be improved through genetic modification, only a few reports have described the application of the in *S*. *cerevisiae* biosynthesis pathway for aromatic amino acids for chemical production [15, 16, 21].

To demonstrate that the genomes of herbaceous biomass such as *B*. *distachyon* is a versatile and useful resource for genes involved in the production of aromatic compounds, here, we searched the *B*. *distachyon* genome for genes encoding L-tyrosine decarboxylase (TDC), which is involved in alkaloid biosynthesis. In *E*. *coli*, tyramine production pathway was previously reported, and TDC gene derived from *Lactobacillus brevis* JCM1170 was used for tyramine production in that report [22]. Several genes annotated as TDC encoding were identified by screening *B*. *distachyon* cDNA libraries and were then evaluated using *S*. *cerevisiae* as a host. TDC-expressing *S*. *cerevisiae* successfully converted L-tyrosine to tyramine, which is the decarboxylation product of L-tyrosine. By increasing L-tyrosine availability, tyramine productivity by the recombinant *S*. *cerevisiae* strain expressing TDC derived from *B*. *distachyon* was 6.6-fold higher than that of the control strain.

## Materials and Methods

### Plasmid construction and yeast transformation

Polymerase chain reactions (PCR) were performed using PrimeSTAR HS (Takara Bio, Shiga, Japan) and the primer pairs listed in Table 1. PCR cycle conditions were as follows: 98°C for 1 minute followed by 30 cycles of 98°C for 15s, 68°C for 30s, and 72°C for 90s. Plasmids for transformation of *S*. *cerevisiae* were constructed by PCR amplifying the identified gene fragments encoding TDC homologs using Bradi1g28960.1, Bradi2g51120.1, Bradi2g51170.1, Bradi3g14750.1, or Bradi3g14780.1 as a template with the appropriate primer pairs. Each gene was identified using GRAMENE (http://www.gramene.org/) (Brachypodium.org is also available (http://www.brachypodium.org/)). PCR cycle conditions were as follows: 98°C for 1 minute followed by 30 cycles of 98°C for 15s, 68°C for 30s, and 72°C for 90s. Each amplified fragment was introduced into the *Nhe*I or *Sal*I, and *Xma*I sites of pGK422 [21], generating plasmids pGK422-*tdc60*, pGK422-*tdc20*, pGK422-*tdc70*, pGK422-*tdc50*, or pGK422-*tdc80*. δ-integrative plasmids were constructed by PCR amplifying the gene fragment encoding *LEU2* from pRS405 DNA [22] with LEU2d(F)_InF and LEU2d(R)_InF. The obtained fragment was introduced into the *Xho*I sites of pδU [23], which contained URA3 as a selective marker, using an In-Fusion HD Cloning kit (Takara Bio), generating the plasmid pδL. A gene fragment containing the *PGK1* promoter region was amplified by PCR using pGK422 as a template with the appropriate primer pair and was then introduced into the *Pst*I and *Bam*HI sites of pδU and pδL using an In-Fusion HD Cloning kit, generating pδU-PGK and pδL-PGK, respectively. The synthetic gene fragments *ARO4* ^*fbr*^ and *ARO7* ^*fbr*^ were obtained from a commercial source (Invitrogen, San Diego, CA) (see S1 File). A gene fragment encoding ARO4^fbr^ was PCR amplified using *ARO4* ^*fbr*^ as a template with ARO4^fbr^_Fw and ARO4^fbr^_Rv, and was then introduced into the *Bam*HI sites of pδU-PGK using an In-Fusion HD Cloning kit, generating pδU-*ARO4* ^*fbr*^. The synthetic *ARO7* ^*fbr*^ gene fragment was directly introduced into the *Bam*HI sites of pδL-PGK using an In-Fusion HD Cloning kit, generating pδL-*ARO7* ^*fbr*^.

**Table 1:** Strains, plasmids, transformants, and oligonucleotide primers used in this study.

Strain, plasmid, primer, or transformant	Relevant features	Source or reference
**Strains**		
*Escherichia coli* Nova blue	*endA1 hsdR17*(r_*K12*_^*-*^m_*K12*_^+^) *supE44 thi-I gyrA96 relA1 lac* recA1/F’[proAB+ lacIq ZΔM15::Tn10(Tetr)]; used for gene cloning.	Novagene
*Saccharomyces cerevisiae* YPH499	*MAT* **a** *ura3-52 leu2-Δ1 lys2-801 his-Δ200 trp1-Δ63 ade2-101*	ATCC
**Plasmids**		
pGK422	Versatile vector containing 2μ *ori* (long form, from pWI3) and *PGK1* promoter in *S*. *cerevisiae*; selection marker is *ADE2*.	[23]
pGK422-*tdc20*	Vector for expressing Bradi2g51120.1; created from pGK422	This study
pGK422-*tdc70*	Vector for expressing Bradi2g51170.1; created from pGK422	This study
pGK422-*tdc60*	Vector for expressing Bradi1g28960.1; created from pGK422	This study
pGK422-*tdc50*	Vector for expressing Bradi3g14750.1; created from pGK422	This study
pGK422-*tdc80*	Vector for expressing Bradi3g14780.1; created from pGK422	This study
PδU	δ-integration vector in *S*. *cerevisiae*; selection marker is *URA3*.	[24]
PδL	δ-integration vector in *S*. *cerevisiae*; selection marker is *LEU2*.	This study
pδU-PGK	Versatile δ-integration vector including *PGK1* promoter in *S*. *cerevisiae*; created from pδU.	This study
pδL-PGK	Versatile δ-integration vector including *PGK1* promoter in *S*. *cerevisiae*; created from pδL.	This study
pδU-*ARO4* ^*fbr*^	Vector for expressing *ARO4* ^*fbr*^; created from pδU-PGK	This study
pδL-*ARO7* ^*fbr*^	Vector for expressing *ARO7* ^*fbr*^; created from pδL-PGK	This study
**Transformants**		
YPH499/p422	YPH499 harboring pGK422	This study
YPH499/p422*tdc20*	YPH499 transformant harboring pGK422-*tdc20*	This study
YPH499/p422*tdc70*	YPH499 transformant harboring pGK422-*tdc70*	This study
YPH499/p422*tdc60*	YPH499 transformant harboring pGK422-*tdc60*	This study
YPH499/p422*tdc50*	YPH499 transformant harboring pGK422-*tdc50*	This study
YPH499/p422*tdc80*	YPH499 transformant harboring pGK422-*tdc80*	This study
YPH499/δU/δL	YPH499 transformant integrated pδU-PGK and pδL-PGK.	This study
YPH499/δU*ARO4* ^*fbr*^	YPH499 transformant integrated pδU-*ARO4* ^*fbr*^.	This study
YPH499/δU*ARO4* ^*fbr*^/δL	YPH499 transformant integrated pδU-*ARO4* ^*fbr*^ and pδL-PGK.	This study
YPH499/δU/δL*ARO7* ^*fbr*^	YPH499 transformant integrated pδU-PGK and pδL-*ARO7* ^*fbr*^.	This study
YPH499/δU*ARO4* ^*fbr*^/δL*ARO7* ^*fbr*^	YPH499 transformant integrated pδU-*ARO4* ^*fbr*^ and pδL-*ARO7* ^*fbr*^.	This study
YPH499/δU/δL/*tdc70*	YPH499/δU/δL transformant harboring pGK422-*tdc70*	This study
YPH499/δU*ARO4* ^*fbr*^/δL/*tdc70*	YPH499/δU*ARO4* ^*fbr*^/δL transformant harboring pGK422-*tdc70*	This study
YPH499/δU/δL*ARO7* ^*fbr*^/*tdc70*	YPH499/δU/δL*ARO7* ^*fbr*^ transformant harboring pGK422-*tdc70*	This study
YPH499/δU*ARO4* ^*fbr*^/δL*ARO7* ^*fbr*^/*tdc70*	YPH499/δU*ARO4* ^*fbr*^/δL*ARO7* ^*fbr*^ transformant harboring pGK422-*tdc70*	This study
**Oligonucleotide primers**		
Br28960.1_Fw	AAAAGCTAGCATGCGGCCGATGGACGAGGA	
Br28960.1_Rv	GGTTCCCGGGCTACTGTACAACATTTCCTA	
Br51120.1_Fw	ACGCGTCGACATGGCACCAACGTCGATGTG	
Br51120.1_Rv	TCCCCCCGGGTTAACCAAGCACGCTGTAGA	
Br51170.1_Fw	ACGCGTCGACATGGCCCCACCGTCGCACTT	
Br51170.1_Rv	TCCCCCCGGGTTAACCAAGCACACTGTAGA	
Br14750.1_Fw	AAAAGCTAGCATGGGCAGCATCGACACCAA	
Br14750.1_Rv	GGTTCCCGGGTTAATCCATCATCTCGCTGG	
Br14780.1_Fw	AAAAGCTAGCATGGGCAGCCTCGACTCGAC	
Br14780.1_Rv	AATTCCCGGGCTAGTGCTCCGCTTCCTCTA	
LEU2d(F)_InF	ATCGATACCGTCGACCTCGAGACGTTGAGCCATTAGTATCAATTTG	
LEU2d(R)_InF	GGTACCGGGCCCCCCCTCGAGTTTACATTTCAGCAATATATATATA	
PGK_to_delta_Fw	CTTGATATCGAATTCCTCGAGAAAGATGCCGATTT	
PGK_to_delta_Rv	CGCTCTAGAACTAGTAGCTTTAACGAACGCAGAAT	
ARO4^fbr^_Fw	AGCGTCGACACTAGTATGAGTGAATCTCCAATGTT	
ARO4^fbr^_Rv	TTCTCTAGACCCGGGTCATTAATGATGGTGATGATGATGTTTCTTGTTAACTTCTCTTC	
RT_ARO4_Fw	TTGTCATTGTCGGTCCTTGTTC	
RT_ARO4_Rv	CGGTTGTTCTTGGCTTCTCC	
RT_ARO7_Fw	ATGTCCTTCAGTTTATGAGGCAAAC	
RT_ARO7_Rv	TGAAAGAGCCCAATCCAAGAA	
RT_PGK1_Fw	TTGGAGAACCCAACCAGACC	
RT_PGK1_Rv	TGAAAGCCATACCACCACCA	

Plasmids were transformed into *S*. *cerevisiae* using lithium acetate method [24, 25], and the resulting transformants are listed in Table 1. The transformants with the highest tyramine or L-tyrosine productivity were selected and used in subsequent experiments.

### Culture conditions

A single colony of each *S*. *cerevisiae* transformant was inoculated into a test tube containing 5 mL synthetic dextrose (SD) medium containing 2% glucose without adenine, uracil, or leucine as preculture. To evaluate tyramine or L-tyrosine productivity, preculture broth was seeded into 5 mL SD medium containing 2% glucose to give an initial OD_600_ value of 0.1. Test tubes were incubated at 30°C for 72 h with agitation at 180 rpm.

### Analytical methods

The concentration of ethanol and glucose in the culture supernatant was measured using a BF-5 biosensor (Oji Scientific Instruments, Hyogo, Japan).

For estimation of produced L-tyrosine and tyramine, GC-MS was carried out using a GCMS-QP2010 Ultra (Shimadzu, Kyoto, Japan) equipped with a CP-Sil 8 CB-MS capillary column (30 m x 0.25 mm x 0.25 μm; Agilent). Helium was used as carrier gas to maintain a flow rate of 2.1 ml/min. The injection volume was 1 μl with a split ratio of 1:10. The oven temperature was initially held at 150°C for 5 min, raised to 300°C at 10°C/min, and further maintained at 300°C for 5 min. The total running time was 25 min. The other settings were as follows: 250°C interface temperature, 200°C ion source temperature, and electron impact ionization (EI) at 70 eV. Dried residues of tyramine and tyrosine were derivatized for 60 min at 80°C in 50 μL N-(tert-butyldimethylsilyl)-N-methyl-trifluoroacetamide (MTBSTFA) and 50 μL N, N-dimethylformamide prior to analysis [26, 27]. Cycloleucine was used as the internal standard.

### Quantification of integrated copy numbers by real-time PCR

The integrated copy number of each recombinant strain was quantified using real-time PCR. Template genomic DNA was isolated from yeast cells cultivated in SD medium for 72 h at 30°C using a GenTLE precipitation carrier (Takara Bio) following the manufacturer’s protocol. The two sets of PCR primers used to detect *ARO4* and *ARO4* ^*fbr*^, and *ARO7* and *ARO7* ^*fbr*^ listed in Table 1. Quantitative real-time PCR was performed using an ABI PRISM 7000 Sequence Detection System (Applied Biosystems, Foster City, CA) with thunderbird SYBR qPCR Mix (Toyobo, Osaka, Japan). The normalized gene copy number was calculated by the relative quantification method with the *PGK1* gene as the housekeeping gene.

## Results

### Cloning and functional expression of the gene encoding *B*. *distachyon* L-tyrosine decarboxylase in *S*. *cerevisiae*

The *B*. *distachyon* genome was screened for genes homologous to *TDC* genes derived from *A*. *thaliana* using GRAMENE, and 5 candidate *TDC* genes (Bradi1g28960.1, Bradi2g51120.1, Bradi2g51170.1, Bradi3g14750.1, and Bradi3g14780.1) were identified. After cloning each candidate gene into multi-copy vector pGK422, the resulting *TDC* expression vectors were individually introduced into *S*. *cerevisiae* YPH499. Each transformant was cultured in SD medium, and the culture supernatant was analyzed by GC-MS. A specific peak derived from tyramine-tyramine-2TBDMS derivatives (m/z = 144) was observed at approximately 17.2 min in GC-MS spectra of the culture supernatants of YPH499/p422*tdc20* and YPH499/p422*tdc70*, but was not detected in the culture supernatants of the control strain, YPH499/p422, or those of YPH499/p422*tdc60*, YPH499/p422*tdc50*, and YPH499/p422*tdc80* (data not shown). YPH499/p422*tdc20* and YPH499/p422*tdc70* produced 20 and 25 mg/L tyramine, respectively, in medium containing 2% glucose as the carbon source. The results of these analysis demonstrated that the *B*. *distachyon* transcripts Bradi2g51120.1 and Bradi2g51170.1 encoded a gene encoding TDC.

### Construction of a L-tyrosine over-producing *S*. *cerevisiae* strain

To increase tyramine productivity in *S*. *cerevisiae*, we attempted to construct a strain that overproduces L-tyrosine by introduction of the enzymes, ARO4 and ARO7, which regulate L-tyrosine biosynthesis in *S*. *cerevisiae* [19, 20], into YPH499. After the construction of YPH499/δU*ARO4* ^*fbr*^, the gene encoding ARO7^fbr^ was introduced into that transformant. Both *ARO4* and *ARO7* were integrated into the genome of YPH499 using the δ-integration method [24].

**Fig 2:**
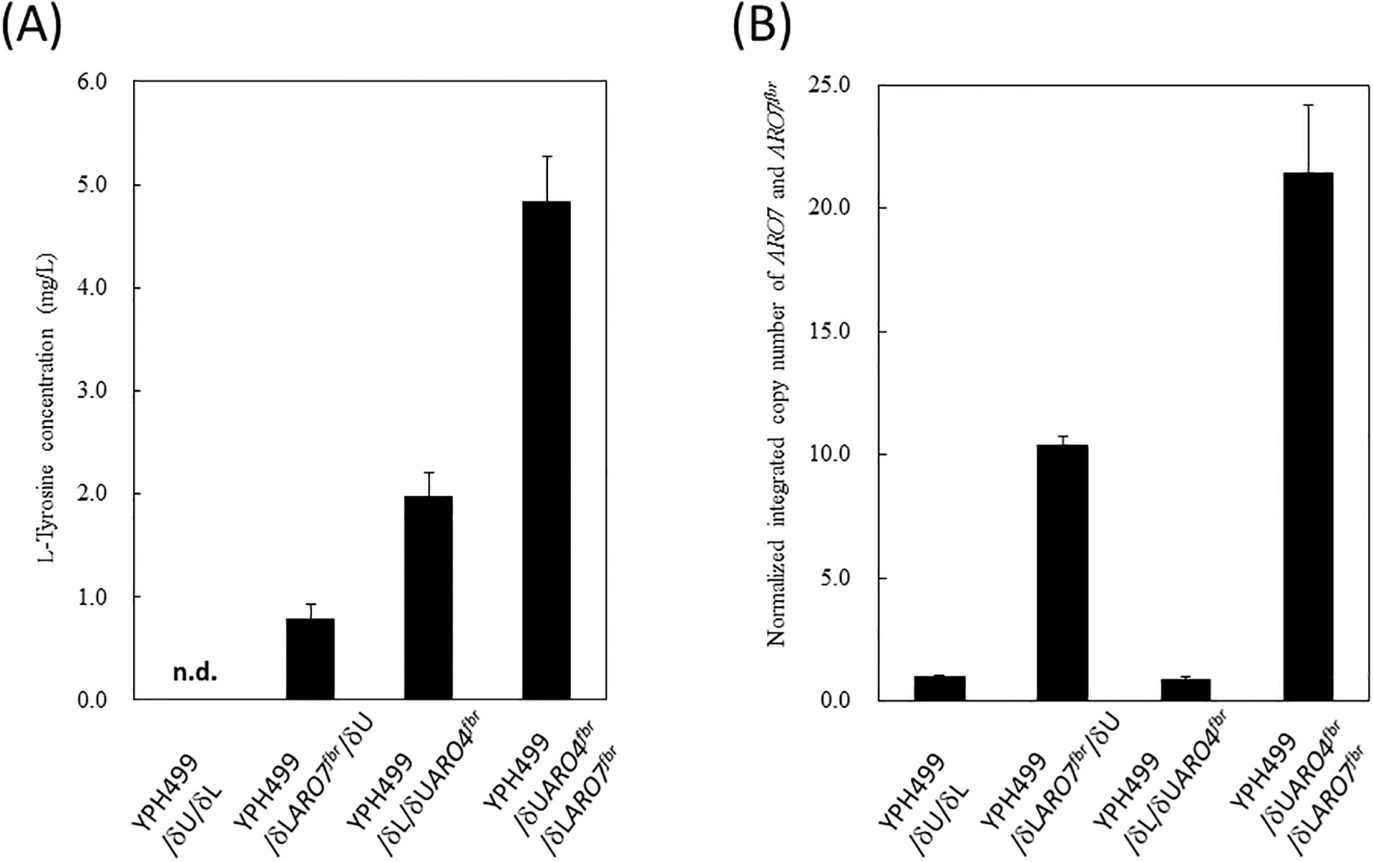
Evaluation of L-tyrosine over-producing *S*. *cerevisiae* constructed in this study. Each bar chart shows the average of 3 independent experiments, and error bars represent the standard deviation. (A) Evaluation of L-tyrosine productivity in the culture supernatants of YPH499/δU/δL, YPH499/δU*ARO4* ^*fbr*^/δL, YPH499/δU/δL*ARO7* ^*fbr*^, and YPH499/δU*ARO4* ^*fbr*^/δL*ARO7* ^*fbr*^. (B) Determination of *ARO7* and *ARO7* ^*fbr*^ gene copy numbers in YPH499/δU/δL, YPH499/δU*ARO4* ^*fbr*^/δL, YPH499/δU/δL*ARO7* ^*fbr*^, and YPH499/δU*ARO4* ^*fbr*^/δL*ARO7* ^*fbr*^ (*ARO4* ^*fbr*^; Ser to Ala substitution in *ARO4* at position 195: Gly to Ser substitution in *ARO7* at position 141).

YPH499/δU/δL, YPH499/δU/δL*ARO7* ^*fbr*^, YPH499/δU*ARO4* ^*fbr*^/δL and YPH499/δU*ARO4* ^*fbr*^/δL*ARO7* ^*fbr*^ were cultured in SD medium containing 2% glucose, and the culture supernatants were analyzed by GC-MS to quantify the amount of L-tyrosine produced after 72 h cultivation (Fig 2A). A total of 0.80, 1.99 and 4.84 mg/L tyrosine was found in the culture supernatant of YPH499/δU/δL*ARO7* ^*fbr*^, YPH499/δU*ARO4* ^*fbr*^/δL and YPH499/δU*ARO4* ^*fbr*^/δL*ARO7* ^*fbr*^, respectively, whereas no tyrosine was detected in the culture supernatant of the control strain YPH499/δU/δL. The copy number of *ARO4* ^*fbr*^ integrated into the genome of YPH499/δU*ARO4* ^*fbr*^/δL and YPH499/δU*ARO4* ^*fbr*^/δL*ARO7* ^*fbr*^, which originated from YPH499/δU*ARO4* ^*fbr*^/δL, was estimated by real-time PCR to be 2 in all strains. In contrast, the copy number of *ARO7* ^*fbr*^ in the genome of YPH499/δU*ARO4* ^*fbr*^/δL*ARO7* ^*fbr*^ was estimated to be approximately 20, whereas that of YPH499/δU/δL*ARO7* ^*fbr*^ was approximately 9 (Fig 2(B)).

### Biosynthesis of tyramine using L-tyrosine over-producing *S*. *cerevisiae*

**Fig 3:**
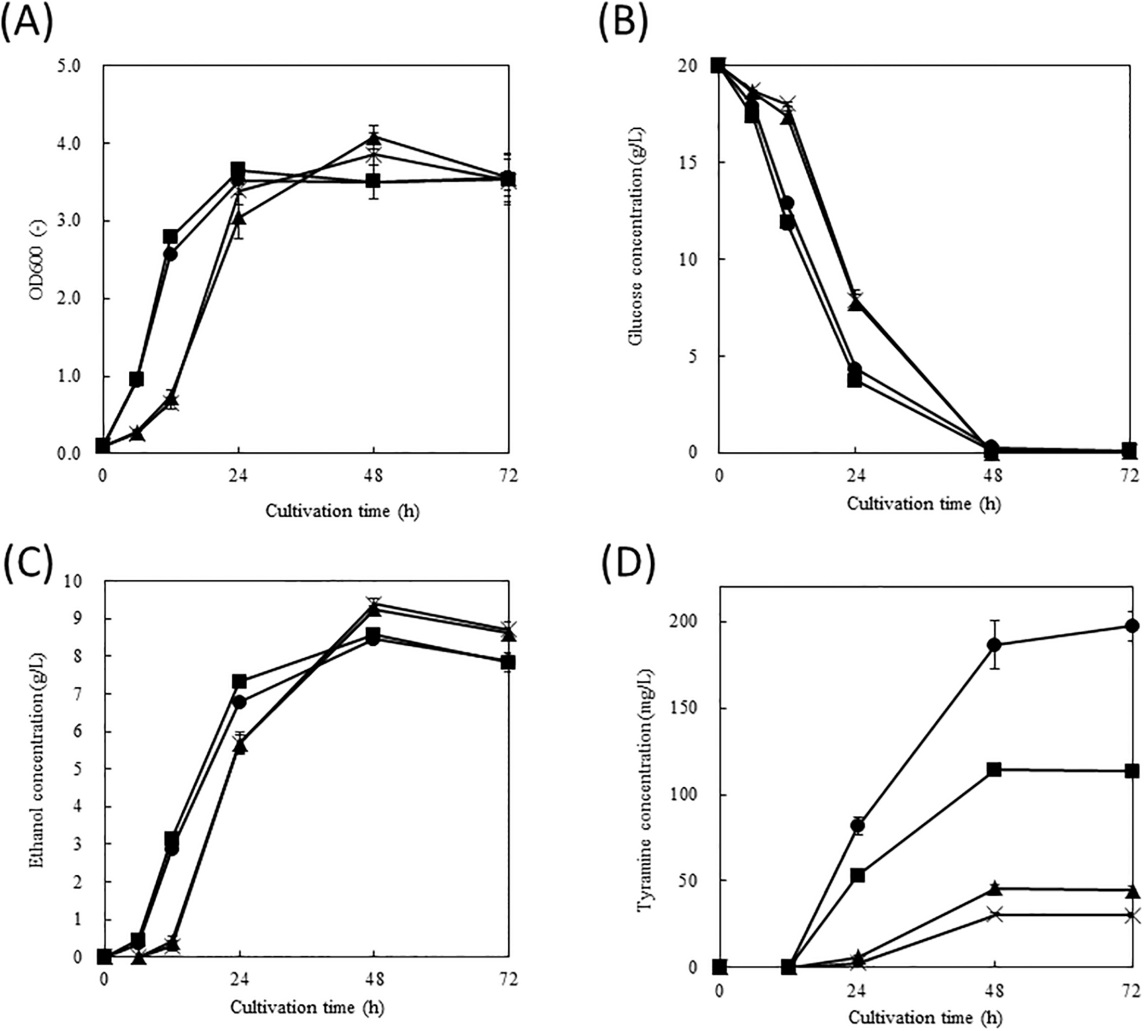
Culture profiles of transformants in SD medium containing 2% glucose as the carbon source. Time-courses of (A) cell growth, (B) glucose consumption, (C) ethanol production, and (D) tyramine production for YPH499/δU/δL/*tdc70* (crosses), YPH499/δU/δL*ARO7* ^*fbr*^/*tdc70* (triangles), YPH499/δU*ARO4* ^*fbr*^/δL/*tdc70* (squares), and YPH499/δU*ARO4* ^*fbr*^/δL*ARO7* ^*fbr*^/*tdc70* (circles). Each data point shows the average of 3 independent experiments, and error bars represent the standard deviation.

To evaluate the ability of *B*. *distachyon* TDC to convert L-tyrosine to tyramine, the gene encoding TDC70 was introduced into strains YPH499/δU/δL, YPH499/δU/δL*ARO7* ^*fbr*^, YPH499/δU*ARO4* ^*fbr*^/δL and YPH499/δU*ARO4* ^*fbr*^/δL*ARO7* ^*fbr*^. Fig 3(A) shows the time courses of cell growth of each transformant. Although the cell growth rates of YPH499/δU*ARO4* ^*fbr*^/δL and YPH499/δU*ARO4* ^*fbr*^/δL*ARO7* ^*fbr*^ were higher than those of YPH499/δU/δL and YPH499/δU/δL*ARO7* ^*fbr*^, the maximal level of cell growth was similar among the four transformants. Fig 3(B) and 3(C) show time courses of the glucose consumption and ethanol production rates, respectively, of each transformant. The rates of glucose consumption and ethanol production of YPH499/δU*ARO4* ^*fbr*^/δL and YPH499/δU*ARO4* ^*fbr*^/δL*ARO7* ^*fbr*^ were higher than those of YPH499/δU/δL and YPH499/δU/δL*ARO7* ^*fbr*^. Fig 3(D) shows the time courses of tyramine production by the recombinant strains. The maximal levels of tyramine production, which started after 12 h cultivation, reached by YPH499/δU/δL, YPH499/δU/δL*ARO7* ^*fbr*^, YPH499/δU*ARO4* ^*fbr*^/δL and YPH499/δU*ARO4* ^*fbr*^/δL*ARO7* ^*fbr*^ were 30.4, 44.7, 113, and 200 mg/L, respectively, after 72 h of cultivation.

## Discussion

Plants accumulate large numbers of compounds that contain aromatic residues, such as phenylpropanoids, flavonoids, coumarins, and alkaloids, via secondary biosynthesis pathways [1–3, 28–30]. The structural diversity of aromatic compounds produced in plants is realized through sets of enzyme superfamilies, such as oxygenases, ligases, and decarboxylases [6]. For example, *(S)*-norcoclaurine, which is an intermediate of the benzylisoquinoline alkaloid biosynthetic pathway, is synthesized from two molecules containing L-tyrosine modified with hydroxyl groups in the benzene ring through reactions catalyzed by aromatic amino acid decarboxylase and monooxygenase [3]. L-tyrosine derivatives can be converted to various compounds due to the hydroxyl group at the para position, and categorized into important parts in aromatic compounds. As various types of enzymes capable of modifying aromatic residues are found in plants, an increasing number of enzymes involved in the synthesis aromatic compounds will be identified as the genomes sequences of more plants become available.

Recently, the complete genome of *B*. *distachyon* was sequenced and used to construct full-length cDNA libraries [9]. To demonstrate that *B*. *distachyon* is a useful gene resource, we here focused on the *B*. *distachyon* genome for homologs of TDC, which catalyzes the decarboxylation of L-tyrosine and is involved in the production of aromatic compounds [31, 32]. It is suggestive that the transcripts Bradi2g51120.1 and Bradi2g51170.1 encode enzymes with L-tyrosine decarboxylation activity, and the corresponding genes were identified as novel *TDC* genes of *B*. *distachyon*.

The activity of *B*. *distachyon* TDC was further evaluated by constructing an L-tyrosine over-producing strain of *S*. *cerevisiae*. In the biosynthesis pathway of L-tyrosine in *S*. *cerevisiae*, ARO4 and ARO7 (ARO4^fbr^ and ARO7^fbr^) are key enzymes that regulate L-tyrosine productivity and are subject to feedback inhibition by the produced L-tyrosine [19, 20]. Here, genes encoding L-tyrosine-insensitive ARO4 and ARO7 mutants were introduced into the genome of *S*. *cerevisiae* YPH499 strain using the δ-integration method. Helmstaedt *et al*. reported L-tyrosine-insensitive ARO4^fbr^ [20], whereas ARO7^fbr^ was previously constructed by Luttik et al. [19]. As shown in Fig 2(A), the L-tyrosine productivity of YPH499/δU*ARO4* ^*fbr*^/δL and YPH499/δU/δL*ARO7* ^*fbr*^ was higher than that of YPH499/δU/δL. Quantitative real-time PCR analysis revealed that 2 copies of *ARO4* ^*fbr*^ were introduced into the genome of YPH499/δU*ARO4* ^*fbr*^/δL, whereas approximately 10 copies of *ARO7* ^*fbr*^ genes were integrated into the YPH499/δU/δL*ARO7* ^*fbr*^ genome (Fig 2(B)). Together, these findings indicate that ARO4^fbr^ enhances L-tyrosine productivity more efficiently than ARO7^fbr^ (Fig 2(A)). This result may be attributed to the low availability of intracellular chorismate in YPH499/δU/δL*ARO7* ^*fbr*^ compared to that in ARO4^fbr^-expressing strains. ARO4^fbr^ catalyzes the specific condensation of E4P and PEP into chorismate in the first step of the shikimate pathway, and the subsequent dislocation reaction is catalyzed by ARO7^fbr^ (Fig 1). As YPH499/δU/δL*ARO7* ^*fbr*^ expresses L-tyrosine sensitive ARO4, the formation of chorismate is strongly regulated by the produced L-tyrosine, which would therefore limit the available chorismate in this strain. Consistent with this speculation, the amount L-tyrosine produced by YPH499/δU*ARO4* ^*fbr*^/δL*ARO7* ^*fbr*^ reached 4.84 mg/L in the culture supernatant, whereas YPH499/δU/δL did not produce L-tyrosine at detectable levels. We also investigated the correlation between L-tyrosine productivity and the copy number of *ARO4* ^*fbr*^ or *ARO7* ^*fbr*^. Although the copy number of *ARO4* ^*fbr*^ affected L-tyrosine productivity in the case of *ARO4* ^*fbr*^ (See S2 File), L-tyrosine productivity wasn’t proportional to the copy number of *ARO7* ^*fbr*^ in the case of *ARO7* ^*fbr*^ (See S3 File).

The TDC homolog of *B*. *distachyon* encoded by Bradi2g51170.1 was functionally characterized by introduction into YPH499/δU*ARO4* ^*fbr*^/δL*ARO7* ^*fbr*^. YPH499/δU*ARO4* ^*fbr*^/δL*ARO7* ^*fbr*^/*tdc70* was cultured using SD medium containing 2% glucose as the carbon source. As shown in Fig 3(D), 200 mg/L tyramine was produced by YPH499/δU*ARO4* ^*fbr*^/δL*ARO7* ^*fbr*^/*tdc70*, a level that was 6.6-fold higher than that of YPH499/δU/δL/*tdc70* as the control strain. Based on the cell density, and protein and amino acid compositions of *S*. *cerevisiae*, we estimated the flux distribution rates of L-tyrosine into tyramine and biomass in each transformant after 72 h of cultivation [33, 34]. With increasing L-tyrosine productivity, the ratio of L-tyrosine distributed into tyramine was increased (Table 2). As L-tyrosine was not detected in the culture supernatant of the tyramine-producing strains, free L-tyrosine was thought to be completely converted to tyramine. These findings indicate that one of the rate-limiting steps of tyramine production remains L-tyrosine availability. Thus, the TDC encoded by Bradi2g51170.1 may be a promising enzyme for the microbial production of aromatic compounds. We also attempted to express a candidate TDC derived from *A*. *thaliana* in *S*. *cerevisiae*; however, *A*. *thaliana* TDC could not be expressed using our expression system (data not shown). As shown in Fig 3(A)–3(C), the cell growth, glucose consumption, and ethanol production rates of YPH499/δU*ARO4* ^*fbr*^/δL*ARO7* ^*fbr*^/*tdc70* and YPH499/δU*ARO4* ^*fbr*^/δL/*tdc70* were higher than those of YPH499/δU/δL/*tdc70* and YPH499/δU/δL*ARO7* ^*fbr*^/*tdc70*. These results may be attributed to the greater carbon flux in the glycolysis pathway resulting from the expression of ARO4^fbr^, which promotes the condensation of PEP and E4P. In this study, we transformed pGK422-*tdc70* into two different *ARO4* ^*fbr*^- and *ARO7* ^*fbr*^-expressing backgrounds. As a result, tyramine productivity, cell growth rates, glucose consumption rate, and ethanol production rates were almost the same among them (See S4 File). Using YPH499/δU*ARO4* ^*fbr*^ as the parent strain, TDC20 was also evaluated and compared to TDC70. Tyramine productivity of TDC70 was slightly higher than that of TDC20 (See S5 File).

**Table 2:** Flux distribution of L-tyrosine produced in each transformant to tyramine and biomass (all produced L-tyrosine was considered to be converted to tyramine except for the proportion incorporated into biomass).

	YPH499/δU/δL/*tdc70*	YPH499/δU/δL*ARO7* ^*fbr*^/*tdc70*	YPH499/δU*ARO4* ^*fbr*^/δL/*tdc70*	YPH499/δU*ARO4* ^*fbr*^/δL*ARO7* ^*fbr*^/*tdc70*
Tyramine (mol%)	51.9 ±1.8	61.1 ±2.3	80.0 ±1.8	87.4 ±0.3
Biomass* (mol%)	48.1 ±1.8	38.9 ±2.3	20.0 ±1.8	12.6 ±0.3

*A flux value to tyrosine building biomass was determined from OD_600_ values and its conversion coefficient to dry cell weight (0.25 g-DCW/L/OD_600_) by using the composition ratio of L-tyrosine in biomass [33, 34]. The flux was estimated as tyrosine concentration of culture (mmol/L). Flux distributions between tyramine and biomass from tyrosine were estimated from each concentration.

In conclusion, we screened the genome of *B*. *distachyon* for genes encoding TDC, which is an enzyme involved in the modification of aromatic compounds, and identified two putative genes encoding TDC using *S*. *cerevisiae* as a host strain. This result implies that *B*. *distachyon* has high potential as a genetic resource for the microbial production of aromatic compounds. Although aromatic compounds have reportedly been produced using *S*. *cerevisiae*, the yield of L-tyrosine derivatives, such as alkaloids, was very low [21]. We speculate that the L-tyrosine over-producing strain constructed here may be applicable to the production of L-tyrosine derivatives with complicated structures.

## Supporting Information

S1 File The nucleotide sequences of synthetic *ARO4* ^*fbr*^ and *ARO7* ^*fbr*^ genes (Under lines indicate open reading frame, capital letters indicate the nucleotide sequences substituted in order to deregulate feedback inhibition, and italic characters indicate flag-tag sequence). (DOCX)

S2 File Correlation between L-tyrosine productivity and the copy number of *ARO4* ^*fbr*^. YPH499/δU*ARO4^fbr^*/δL (Y; YPH499 (control), 1; colony 6, 2; colony 8, 3; colony 9).1 copy number of *ARO4^fbr^* was integrated into the genome of colony 6 and 8, whereas 2 were colony 9, which was adopted for further experiments in this study.
(DOCX)

S3 File Correlation between L-tyrosine productivity and the copy number of *ARO7* ^*fbr*^. Results of YPH499/δU*ARO4^fbr^*/δL*ARO7^fbr^* (Y; YPH499/δU*ARO4^fbr^*/δL (control), 1; colony 3, 2; colony 5, 3; colony 18, 4; colony adopted in this study). Gray bar indicates L-tyrosine productivity per OD_600_, and black bar indicates normalized integrated copy number of *ARO7* and *ARO7* ^*fbr*^.
(DOCX)

S4 File Culture profiles of transformants in SD medium containing 2% glucose as the carbon source. Time-courses of (A) cell growth, (B) glucose consumption, (C) ethanol production, and (D) tyramine production for YPH499/δU*ARO4* ^*fbr*^/δL*ARO7* ^*fbr*^/*tdc70* adopted in the manuscript (closed circles) and YPH499/δU*ARO4* ^*fbr*^/δL*ARO7* ^*fbr*^/*tdc70* originated from different ARO4/ARO7 background (open circles). Each data point shows the average of 3 independent experiments, and error bars represent the standard deviation.
(DOCX)

S5 File Evaluation of tyramine productivity using YPH499/δU*ARO4* ^*fbr*^/*tdc20* and YPH499/δU*ARO4* ^*fbr*^/*tdc70* after 96 h cultivation. (DOCX)

## References

[1] Winkel-Shirley B. Flavonoid biosynthesis. A colorful model for genetics, biochemistry, cell biology, and biotechnology. Plant Physiol. 2001;126: 485–493. 11402179 10.1104/pp.126.2.485 PMC1540115

[2] Gray J, Caparrós-Ruiz D, Grotewold E. Grass phenylpropanoids: regulate before using! Plant Sci. 2012;184: 112–120. 10.1016/j.plantsci.2011.12.008 22284715

[3] Kutchan TM. Heterologous expression of alkaloid biosynthetic genes—a review. Gene. 1996;179: 73–81. 8955631 10.1016/s0378-1119(96)00426-x

[4] Kärkönen A, Koutaniemi S. Lignin biosynthesis studies in plant tissue cultures. J Integr Plant Biol. 2010;52: 176–185. 10.1111/j.1744-7909.2010.00913.x 20377679

[5] Davin LB, Lewis NG. Lignin primary structures and dirigent sites. Curr Opin Biotechnol. 2005;16: 407–415. 16023847 10.1016/j.copbio.2005.06.011

[6] Vogt T. Phenylpropanoid biosynthesis. Mol Plant. 2010;3: 2–20. 10.1093/mp/ssp106 20035037

[7] Yazaki K, Sasaki K, Tsurumaru Y. Prenylation of aromatic compounds, a key diversification of plant secondary metabolites. Phytochemistry 2009;70: 1739–1745. 10.1016/j.phytochem.2009.08.023 19819506

[8] Mochida K, Shinozaki K. Unlocking Triticeae genomics to sustainably feed the future. Plant Cell Physiol. 2013;54: 1931–1950. 10.1093/pcp/pct163 24204022 PMC3856857

[9] Mochida K, Uehara-Yamaguchi Y, Takahashi F, Yoshida T, Sakurai T, Kazuo Shinozaki. Large-scale collection and analysis of full-length cDNAs from *Brachypodium distachyon* and integration with Pooideae sequence resources. PLoS One. 2013;8: e75265 10.1371/journal.pone.0075265 24130698 PMC3793998

[10] Jiang H, Wood KV, Morgan JA. Metabolic engineering of the phenylpropanoid pathway in *Saccharomyces cerevisiae* . Appl Environ Microbiol. 2005;71: 2962–2969. 15932991 10.1128/AEM.71.6.2962-2969.2005 PMC1151809

[11] Kirby J, Romanini DW, Paradise EM, Keasling JD. Engineering triterpene production in *Saccharomyces cerevisiae*-beta-amyrin synthase from *Artemisia annua* . FEBS J. 2008;275: 1852–1859. 10.1111/j.1742-4658.2008.06343.x 18336574

[12] Westfall PJ, Pitera DJ, Lenihan JR, Eng D, Woolard FX, Regentin R, et al Production of amorphadiene in yeast, and its conversion to dihydroartemisinic acid, precursor to the antimalarial agent artemisinin. Proc Natl Acad Sci U S A 2012;109: E111–118. 10.1073/pnas.1110740109 22247290 PMC3271868

[13] Steen EJ, Chan R, Prasad N, Myers S, Petzold CJ, Redding A, et al Metabolic engineering of *Saccharomyces cerevisiae* for the production of n-butanol. Microb Cell Fact 2008;7: 36–43. 10.1186/1475-2859-7-36 19055772 PMC2621116

[14] Chen Y, Bao J, Kim IK, Siewers V, Nielsen J. Coupled incremental precursor and co-factor supply improves 3-hydroxypropionic acid production in *Saccharomyces cerevisiae* . Metab Eng. 2014;22: 104–109. 10.1016/j.ymben.2014.01.005 24502850

[15] Kim B, Cho BR, Hahn JS. Metabolic engineering of *Saccharomyces cerevisiae* for the production of 2-phenylethanol via Ehrlich pathway. Biotechnol Bioeng. 2014;111: 115–124. 10.1002/bit.24993 23836015

[16] Vannelli T, Wei Qi W, Sweigard J, Gatenby AA, Sariaslani FS. Production of p-hydroxycinnamic acid from glucose in *Saccharomyces cerevisiae* and *Escherichia coli* by expression of heterologous genes from plants and fungi. Metab Eng. 2007;9: 142–151. 17204442 10.1016/j.ymben.2006.11.001

[17] Koopman F, Beekwilder J, Crimi B, van Houwelingen A, Hall RD, Bosch D, et al De novo production of the flavonoid naringenin in engineered *Saccharomyces cerevisiae* . Microb Cell Fact. 2012;11: 155–169 10.1186/1475-2859-11-155 23216753 PMC3539886

[18] Gosset G. Production of aromatic compounds in bacteria. Curr Opin Biotechnol. 2009;20: 651–658. 10.1016/j.copbio.2009.09.012 19875279

[19] Luttik MA, Vuralhan Z, Suir E, Braus GH, Pronk JT, Daran JM. Alleviation of feedback inhibition in *Saccharomyces cerevisiae* aromatic amino acid biosynthesis: quantification of metabolic impact. Metab Eng. 2008;10: 141–153. 10.1016/j.ymben.2008.02.002 18372204

[20] Helmstaedt K, Strittmatter A, Lipscomb WN, Braus GH. Evolution of 3-deoxy-D-arabino-heptulosonate-7-phosphate synthase-encoding genes in the yeast *Saccharomyces cerevisiae* . Proc Natl Acad Sci U S A. 2005;102: 9784–9789. 15987779 10.1073/pnas.0504238102 PMC1175010

[21] Minami H, Kim JS, Ikezawa N, Takemura T, Katayama T, Kumagai H, et al Microbial production of plant benzylisoquinoline alkaloids. Proc Natl Acad Sci U S A. 2008;105: 7393–7398. 10.1073/pnas.0802981105 18492807 PMC2396723

[22] Koma D, Yamanaka H, Moriyoshi K, Ohmoto T, Sakai K. A convenient method for multiple insertions of desired genes into target loci on the Escherichia coli chromosome. Appl Microbiol Biotechnol. 2012;93: 815–829. 10.1007/s00253-011-3735-z 22127754

[23] Ishii J, Izawa K, Matsumura S, Wakamura K, Tanino T, Ogino C, et al A simple and immediate method for simultaneously evaluating expression level and plasmid maintenance in yeast. J Biochem. 2009;145: 701–708. 10.1093/jb/mvp028 19237442

[24] Yamada R, Tanaka T, Ogino C, Fukuda H, Kondo A. Novel strategy for yeast construction using delta-integration and cell fusion to efficiently produce ethanol from raw starch. Appl Microbiol Biotechnol. 2010;85: 1491–1498. 10.1007/s00253-009-2198-y 19707752

[25] Chen DC, Yang BC, Kuo TT. One-step transformation of yeast in stationary phase. Curr Genet. 1992;21:83–84. 1735128 10.1007/BF00318659

[26] Mawhinney TP, Robinett RS, Atalay A, Madson MA. Analysis of amino acids as their tert.-butyldimethylsilyl derivatives by gas-liquid chromatography and mass spectrometry. J Chromatogr. 1986;358: 231–242. 3722299 10.1016/s0021-9673(01)90333-4

[27] MacKenzie SL, Tenaschuk D, Fortier G. Analysis of amino acids by gas-liquid chromatography as tert.-butyldimethylsilyl derivatives. Preparation of derivatives in a single reaction. J Chromatogr. 1987;387: 241–253. 3558623 10.1016/s0021-9673(01)94528-5

[28] Olsen KM, Lea US, Slimestad R, Verheul M, Lillo C. Differential expression of four Arabidopsis PAL genes; PAL1 and PAL2 have functional specialization in abiotic environmental-triggered flavonoid synthesis. J Plant Physiol. 2008;165: 1491–1499. 10.1016/j.jplph.2007.11.005 18242769

[29] Chen H, Jiang H, Morgan JA. Non-natural cinnamic acid derivatives as substrates of cinnamate 4-hydroxylase. Phytochemistry. 2007;68: 306–311. 17141284 10.1016/j.phytochem.2006.10.018

[30] Wanner LA, Li G, Ware D, Somssich IE, Davis KR. The phenylalanine ammonia-lyase gene family in *Arabidopsis thaliana* . Plant Mol Biol. 1995;27: 327–338. 7888622 10.1007/BF00020187

[31] Lan X, Chang K, Zeng L, Liu X, Qiu F, Zheng W, et al Engineering salidroside biosynthetic pathway in hairy root cultures of *Rhodiola crenulata* based on metabolic characterization of tyrosine decarboxylase. PLoS One. 2013;8: e75459 10.1371/journal.pone.0075459 24124492 PMC3790822

[32] Lehmann T, Pollmann S. Gene expression and characterization of a stress-induced tyrosine decarboxylase from *Arabidopsis thaliana* . FEBS Lett. 2009;583: 1895–1900. 10.1016/j.febslet.2009.05.017 19450582

[33] Stephanopoulos G, Aristidou A, Nielsen J. Metabolic Engineering: Principles and Methodologies. Academic Press. 1998;p.68.

[34] Verduyn C, Postma E, Scheffers WA, van Dijken JP. Energetics of *Saccharomyces cerevisiae* in anaerobic glucose-limited chemostat cultures. J Gen Microbiol. 1990;136: 405–412. 2202777 10.1099/00221287-136-3-405

