# Supplementary material for: Evaluation of Brachypodium distachyon L-Tyrosine Decarboxylase Using L-Tyrosine Over-Producing Saccharomyces cerevisiae
Source: PLoS One. 2015 May 21;10(5):e0125488. doi: 10.1371/journal.pone.0125488 (PMC4440718; doi:10.1371/journal.pone.0125488)
Supplement: S1 File — (DOCX) [file pone.0125488.s001.docx]

**File S1**

The nucleotide sequences of synthetic *ARO4^fbr^* and *ARO7^fbr^* genes (Under lines indicate open reading frame, capital letters indicate the nucleotide sequences substituted in order to deregulate feedback inhibition, and italic characters indicate flag-tag sequence).

(A) *ARO4^fbr^* gene (Ser to Ala substitution in *ARO4* at position 195)

tttttatgagtgaatctccaatgttcgctgccaacggcatgccaaaggtaaatcaaggtgctgaagaagatgtcagaattttaggttacgacccattagcttctccagctctccttcaagtgcaaatcccagccacaccaacttctttggaaactgccaagagaggtagaagagaagctatagatattattaccggtaaagacgacagagttcttgtcattgtcggtccttgttccatccatgatctagaagccgctcaagaatacgctttgagattaaagaaattgtcagatgaattaaaaggtgatttatccatcattatgagagcatacttggagaagccaagaacaaccgtcggctggaaaggtctaattaatgaccctgatgttaacaacactttcaacatcaacaagggtttgcaatccgctagacaattgtttgtcaacttgacaaatatcggtttgccaattggttctgaaatgcttgataccatttctcctcaatacttggctgatttggtctccttcggtgccattggtgccagaaccaccgaatctcaactgcacagagaattggcctccggtttgGCTttcccagttggtttcaagaacggtaccgatggtaccttaaatgttgctgtggatgcttgtcaagccgctgctcattctcaccatttcatgggtgttactaagcatggtgttgctgctatcaccactactaagggtaacgaacactgcttcgttattctaagaggtggtaaaaagggtaccaactacgacgctaagtccgttgcagaagctaaggctcaattgcctgccggttccaacggtctaatgattgactactctcacggtaactccaataaggatttcagaaaccaaccaaaggtcaatgacgttgtttgtgagcaaatcgctaacggtgaaaacgccattaccggtgtcatgattgaatcaaacatcaacgaaggtaaccaaggcatcccagccgaaggtaaagccggcttgaaatatggtgtttccatcactgatgcttgtataggttgggaaactactgaagacgtcttgaggaaattggctgctgctgtcagacaaagaagagaagttaacaagaaatagttttt

(B) *ARO7^fbr^* gene (Gly to Ser substitution in *ARO7* at position 141)

tttagcgtcgacactagtatggatttcacaaaaccagaaactgttttaaatctacaaaatattagagatgaattagttagaatggaggattcgatcatcttcaaatttattgagaggtcgcatttcgccacatgtccttcagtttatgaggcaaaccatccaggtttagaaattccgaattttaaaggatctttcttggattgggctctttcaaatcttgaaattgcgcattctcgcatcagaagattcgaatcacctgatgaaactcccttctttcctgacaagattcagaaatcattcttaccgagcattaactacccacaaattttggcgccttatgccccagaagttaattacaatgataaaataaaaaaagtttatattgaaaagattataccattaatttcgaaaagagatggtgatgataagaataacttcTCAtctgttgccactagagatatagaatgtttgcaaagcttgagtaggagaatccactttggcaagtttgttgctgaagccaagttccaatcggatatcccgctatacacaaagctgatcaaaagtaaagatgtcgaggggataatgaagaatatcaccaattctgccgttgaagaaaagattctagaaagattaactaagaaggctgaagtctatggtgtggaccctaccaacgagtcaggtgaaagaaggattactccagaatatttggtaaaaatttataaggaaattgttatacctatcactaaggaagttgaggtggaatacttgctaagaaggttggaagag*gattacaaggatgatgacgataaa*taatgacccgggtctagagaattt
