# Supplementary material for: Evaluation of Brachypodium distachyon L-Tyrosine Decarboxylase Using L-Tyrosine Over-Producing Saccharomyces cerevisiae
Source: PLoS One. 2015 May 21;10(5):e0125488. doi: 10.1371/journal.pone.0125488 (PMC4440718; doi:10.1371/journal.pone.0125488)

**File S2**

Correlation between L-tyrosine productivity and the copy number of *ARO4^fbr^*. YPH499/δU*ARO4^fbr^*/δL (Y; YPH499 (control), 1; colony 6, 2; colony 8, 3; colony 9).

1 copy number of *ARO4^fbr^* was integrated into the genome of colony 6 and 8, whereas 2 were colony 9, which was adopted for further experiments in this study.


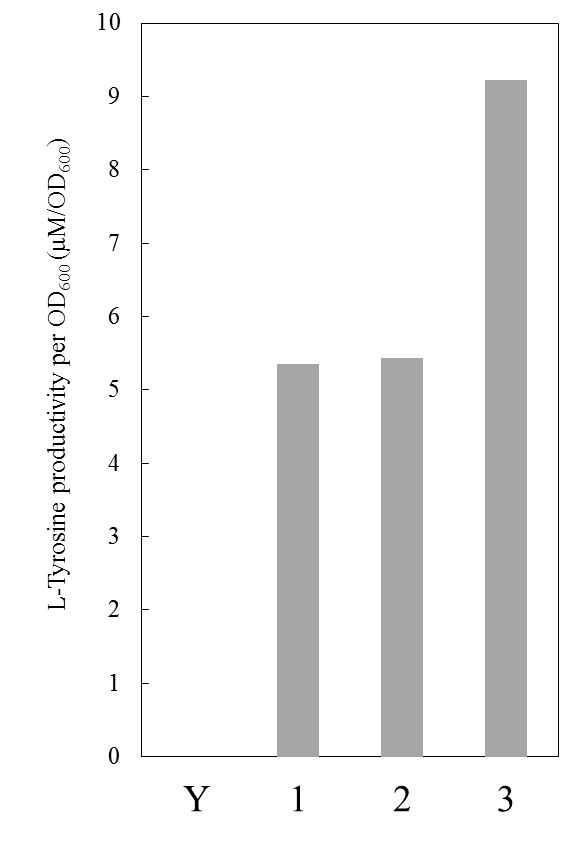

Supplement: S2 File — YPH499/δUARO4fbr/δL (Y; YPH499 (control), 1; colony 6, 2; colony 8, 3; colony 9).1 copy number of ARO4fbr was integrated into the genome of colony 6 and 8, whereas 2 were colony 9, which was adopted for further experiments in this study. (DOCX) [file pone.0125488.s002.docx]
