# Supplementary material for: Evaluation of Brachypodium distachyon L-Tyrosine Decarboxylase Using L-Tyrosine Over-Producing Saccharomyces cerevisiae
Source: PLoS One. 2015 May 21;10(5):e0125488. doi: 10.1371/journal.pone.0125488 (PMC4440718; doi:10.1371/journal.pone.0125488)

**File S3**

Correlation between L-tyrosine productivity and the copy number of *ARO7^fbr^*. Results of YPH499/δU*ARO4^fbr^*/δL*ARO7^fbr^* (Y; YPH499/δU*ARO4^fbr^*/δL (control), 1; colony 3, 2; colony 5, 3; colony 18, 4; colony adopted in this study). Gray bar indicates L-tyrosine productivity per OD_600_, and black bar indicates normalized integrated copy number of *ARO7* and *ARO7^fbr^*.


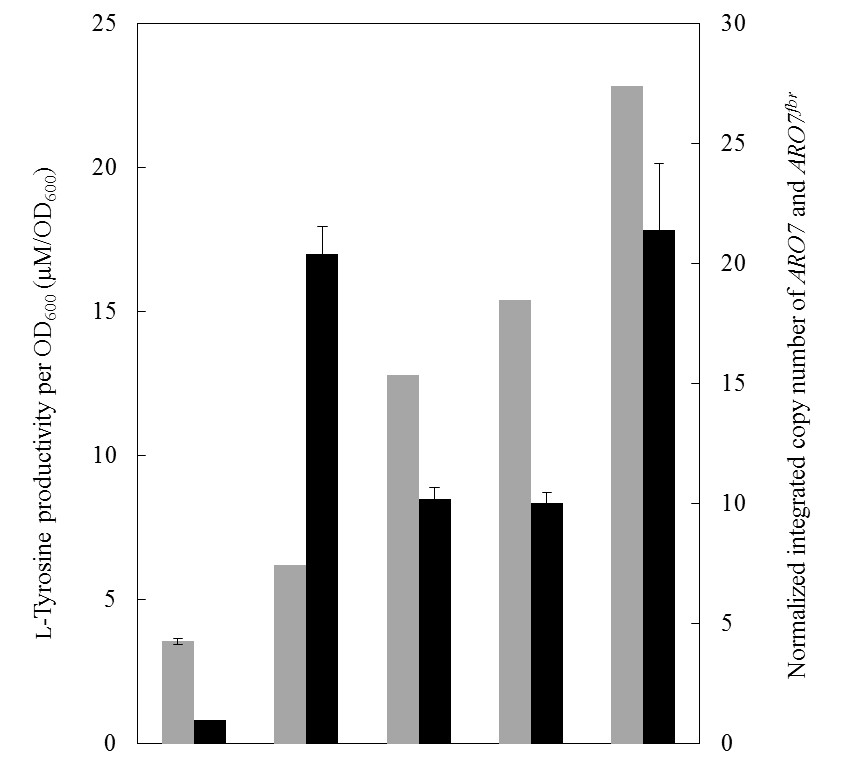

Supplement: S3 File — Results of YPH499/δUARO4fbr/δLARO7fbr (Y; YPH499/δUARO4fbr/δL (control), 1; colony 3, 2; colony 5, 3; colony 18, 4; colony adopted in this study). Gray bar indicates L-tyrosine productivity per OD600, and black bar indicates normalized integrated copy number of ARO7 and ARO7 fbr. (DOCX) [file pone.0125488.s003.docx]
