# Supplementary material for: Evaluation of Brachypodium distachyon L-Tyrosine Decarboxylase Using L-Tyrosine Over-Producing Saccharomyces cerevisiae
Source: PLoS One. 2015 May 21;10(5):e0125488. doi: 10.1371/journal.pone.0125488 (PMC4440718; doi:10.1371/journal.pone.0125488)

**File S4**

Culture profiles of transformants in SD medium containing 2% glucose as the carbon source. Time-courses of (A) cell growth, (B) glucose consumption, (C) ethanol production, and (D) tyramine production for YPH499/δU*ARO4^fbr^*/δL*ARO7^fbr^*/*tdc70* adopted in the manuscript (closed circles) and YPH499/δU*ARO4^fbr^*/δL*ARO7^fbr^*/*tdc70* originated from different ARO4/ARO7 background (open circles). Each data point shows the average of 3 independent experiments, and error bars represent the standard deviation.


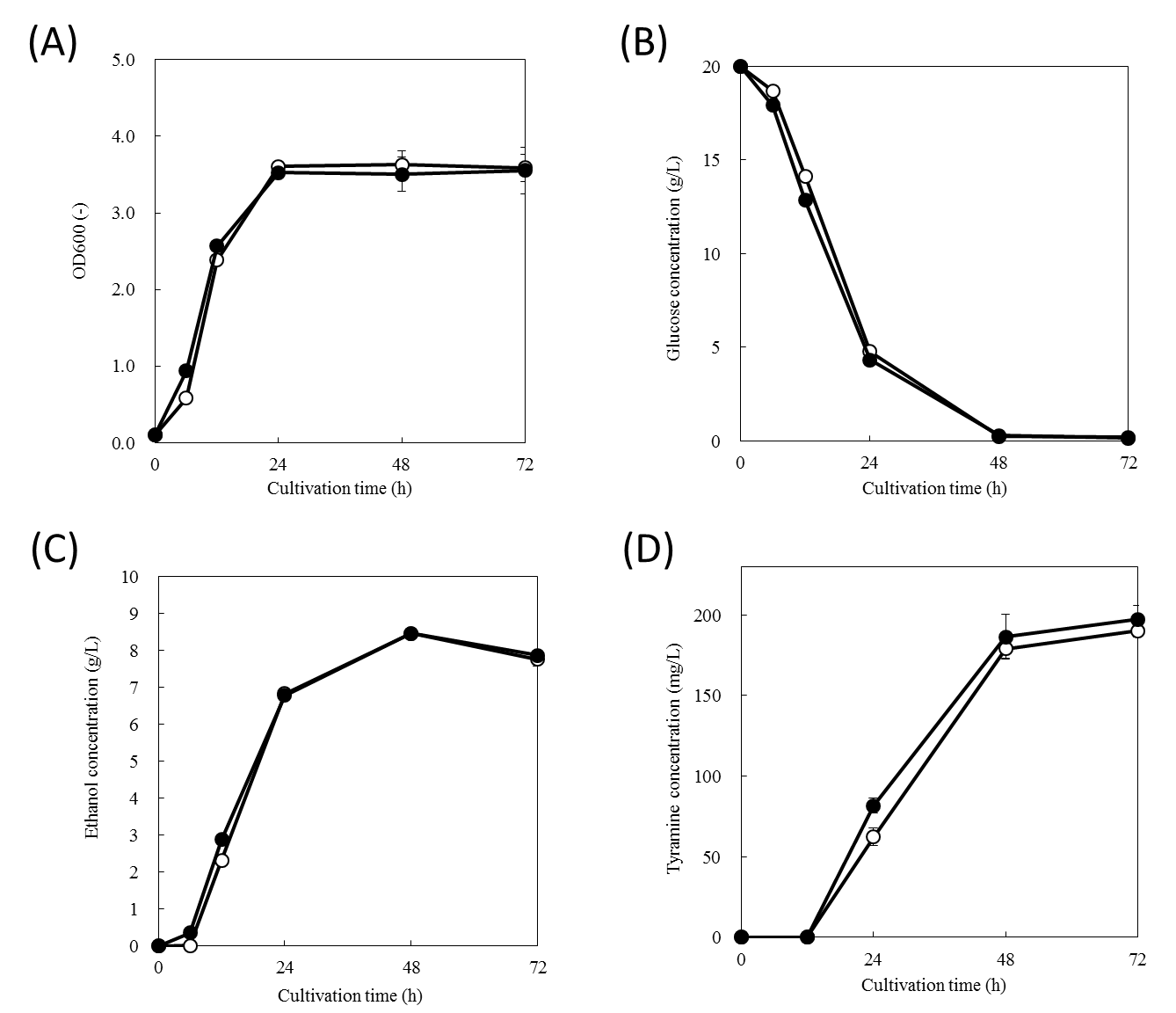

Supplement: S4 File — Time-courses of (A) cell growth, (B) glucose consumption, (C) ethanol production, and (D) tyramine production for YPH499/δUARO4 fbr/δLARO7 fbr/tdc70 adopted in the manuscript (closed circles) and YPH499/δUARO4 fbr/δLARO7 fbr/tdc70 originated from different ARO4/ARO7 background (open circles). Each data point shows the average of 3 independent experiments, and error bars represent the standard deviation. (DOCX) [file pone.0125488.s004.docx]
