# Supplementary material for: Evaluation of Brachypodium distachyon L-Tyrosine Decarboxylase Using L-Tyrosine Over-Producing Saccharomyces cerevisiae
Source: PLoS One. 2015 May 21;10(5):e0125488. doi: 10.1371/journal.pone.0125488 (PMC4440718; doi:10.1371/journal.pone.0125488)

**File S5**

Evaluation of tyramine productivity using YPH499/δU*ARO4^fbr^*/*tdc20* and YPH499/δU*ARO4^fbr^*/*tdc70* after 96 h cultivation.


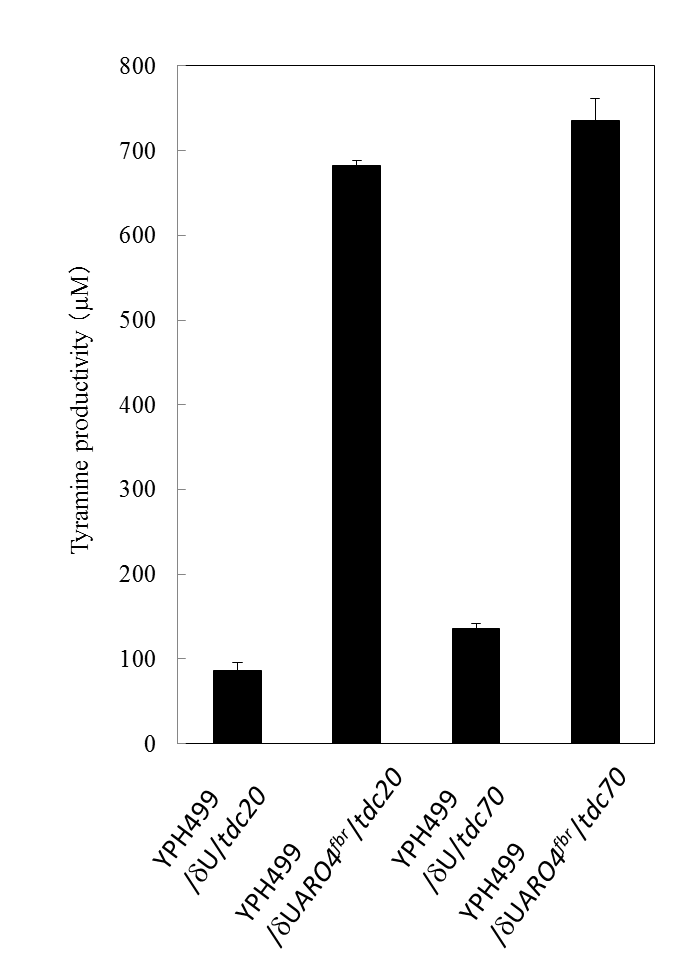

Supplement: S5 File — (DOCX) [file pone.0125488.s005.docx]
